# Parliament and the Profession

**Published:** 1917-06-30

**Authors:** 


### PARLIAMENT AND THE PROFESSION.

### Medical Students.

The Parliamentary Secretary to the Board of Education (Mr. Lewis) informed Mr. MacCallum Scott that the numbers of male and female medical students in medical schools receiving grant from the Board of Education in 1913-14 and 1916-17 are 3,483 and 1,981 respectively. The figures do not include the medical schools at Oxford University, Guy's and Middlesex Hospitals, Bristol University, or the University Colleges of Nottingham, Reading, Southampton, and Cardiff, with regard to which the Board are unable to supply similar information. The Secretary for Scotland stated that the corresponding figures in Scotland are: in the year before the war, 2,367 men and 236 women; in the present year, 1,510 men and 696 women.

### Casualties Amongst Medical Officers.

The Under-Secretary of State for War stated that the numbers of Regular (including temporarily commissioned) and Territorial medical officers recorded in the casualty lists have been as follows: Killed in action, 137; died of wounds, 58; died of disease, 62; wounded, 707. The figures for Colonial officers are not readily available.

### Postage of Saturday Fund Collecting Sheets.

Mr. Crooks, referring to a recent decision under which Hospital Saturday Fund collecting sheets are charged postage under letter rate instead of as printed matter as hitherto, asked if in view of the considerable extra cost to the Fund this decision could be reconsidered. Mr. Illingworth explained the alteration, and while sympathising with the object in view, expressed regret that the Post Office would not be justified in making a distinction in favour of the Saturday Fund.

### Army Nurses Attending Religious Services.

Mr. Ginnell asked the Under-Secretary of State for War whether it is a general rule that all nurses must join in Protestant prayers, and by what authority the superintendent of nurses at Harton Hospital, South Shields, commanded the Catholic girls to attend Protestant prayers on pain of being reported for insubordination. Mr. Macpherson stated that there is no rule of the kind suggested in military hospitals, and that every effort is made to enable Catholic nurses to perform their religious duties. The Harton Hospital, South Shields, is not a military hospital.

